# Geographic and thematic gaps in wildfire resilience research

**DOI:** 10.1016/j.isci.2026.117886

**Published:** 2026-11-06

**Authors:** Caitlin R. Fong, Benjamin S. Halpern

**Affiliations:** 1 National Center for Ecological Analysis and Synthesis, University of California Santa Barbara, Santa Barbara, CA, USA; 2 Bren school of Environmental Science and Management, University of California Santa Barbara, Santa Barbara, CA, USA

**Keywords:** wildfire, resilience, communities, habitats, water, air, carbon, sense of place, infrastructure, livelihoods, biodiversity

## Abstract

Wildfires are becoming more frequent and destructive around the world, threatening lives, homes, and natural landscapes. Building or maintaining systems for wildfire resilience has emerged as a crucial strategy for addressing wildfire risks and impacts, balancing a need for fire on the landscape to support healthy natural systems with a desire to mitigate loss of human life and structure. Our review of over 1,500 wildfire resilience studies shows that while research is growing, it is unevenly distributed across regions and topics, often focusing on habitats while paying less attention to human communities, infrastructure, and other aspects that matter for people’s lives and well-being. By highlighting these gaps, our work helps guide scientists, policymakers, and communities toward more integrated and equitable strategies for living safely with fire. Understanding where research is lacking can improve planning, strengthen resilience efforts, and support decisions that protect both people and ecosystems.

## Highlights

• Reviewed >1,500 publications to assess global patterns in wildfire resilience research
• Research is geographically concentrated in developed, fire-prone regions
• Habitat studies dominate, human dimensions receive less attention, single-method approaches are common
• Cross-domain, multidisciplinary research is needed for equitable wildfire resilience

## Graphical abstract

**Figure:**
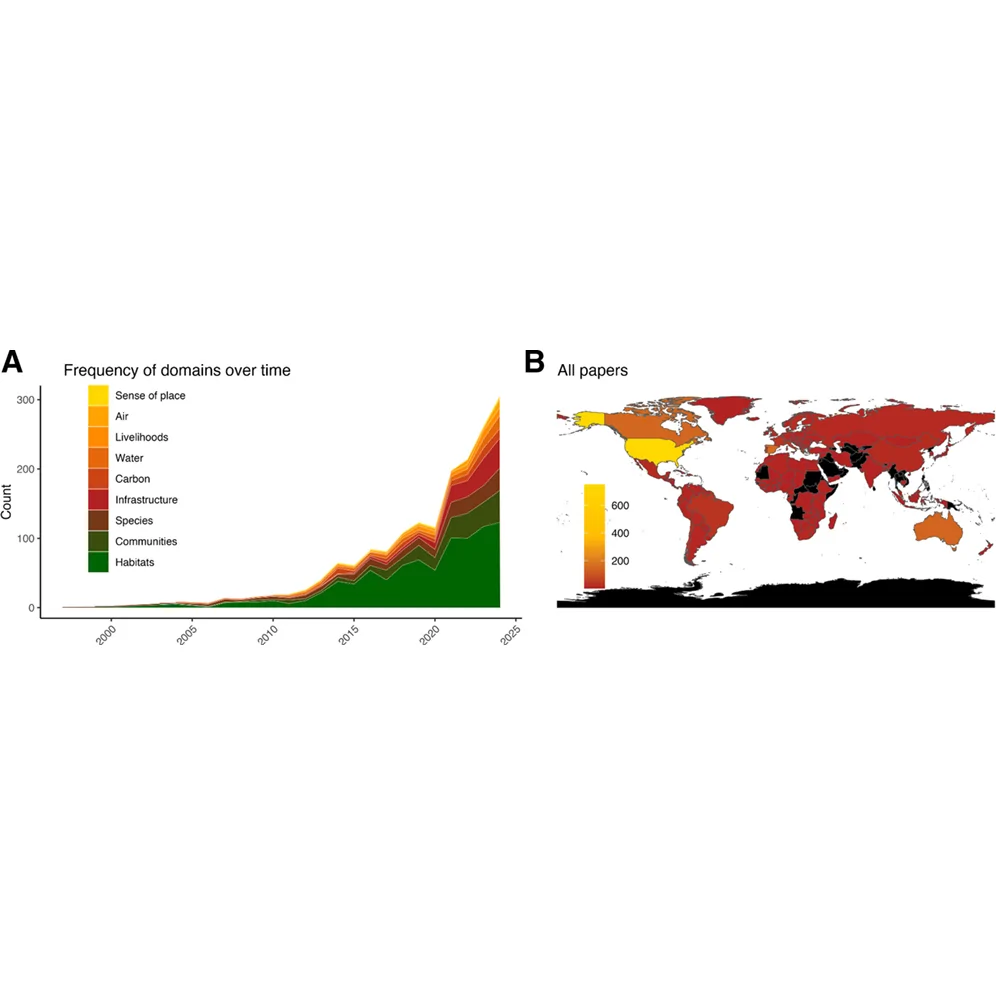

## Teaser

Earth sciences; Environmental science; Environmental management; Environmental monitoring; Environmental policy

## Introduction

Deadly and destructive wildfires have accelerated in pace and scale in the last few decades, motivating scientific and public interest in how to manage natural and built landscapes to be resilient to wildfire. Wildfires are a natural process, with different ecosystems characterized by different fire regimes and natural ranges of variability for fire frequency, intensity, and extent.^1^^,^^2^^,^^3^ Over geologic time scales, ecological communities evolved in the context of fire, and most wildfires fell within the bounds of their evolved states.^1^^,^^4^^,^^5^ However, over the last 100 years, global area burned decreased, a result of fire suppression and land conversion, driving systems out of their natural range of variability.^6^^,^^7^ More recently, extreme wildfires have become more frequent,^8^ and negative effects on social and ecological communities are of global concern.^8^^,^^9^^,^^10^^,^^11^^,^^12^ The magnitude of this challenge may be best exemplified by the record-breaking 2019/2020 wildfire season, when anomalous and catastrophic fires burned across the globe, causing significant loss of life and property and possibly exceeding the bounds of resilience for natural systems.^11^ Yet, wildfire is an inevitable consequence of an oxygenated world—we must learn to live with fire.^5^^,^^13^^,^^14^^,^^15^

Building and maintaining resilience has emerged as a central organizing framework in wildfire policy, management, and planning for confronting the growing challenge of wildfires.^16^^,^^17^^,^^18^ Resilience is a cross-cutting concept that is used in a range of fields, spanning ecology, ecosystem science, political science, business management, disaster and urban planning, and history (reviewed by Martin-Breen P and Anderies JM^19^). While definitions can vary, resilience generally describes the capacity of a system to resist and recover from disturbance within a natural range of variability in state and drivers.^20^^,^^21^^,^^22^ Thus, resilience is a unifying framework that integrates elements of mitigation, adaptation, and transformation while remaining grounded in the dynamics of disturbance and system responses. An erosion of wildfire resilience may be one reason why catastrophic events have become more frequent,^23^ evidenced by the increasing number of catastrophic wildfire events in the last few years.^8^^,^^9^^,^^12^^,^^24^^,^^25^ Further, evidence indicates global climate change will increase wildfire frequency and consequences.^25^^,^^26^^,^^27^^,^^28^ Thus, there is an urgent need to synthesize current understanding of wildfire resilience and identify and address critical knowledge gaps to inform strategies to plan for future wildfire events.

A major part of understanding how wildfire resilience can shape and guide management in understanding how different disciplines and communities use the concept of resilience. To describe the state of knowledge, we evaluated all peer-reviewed literature for how the concept of wildfire resilience is defined, studied, and operationalized, with particular focus on which components of resilience were evaluated. We then categorized each paper by which of nine identified social-ecological domains were addressed (see materials and methods) to evaluate which disciplines are using resilience concepts, where knowledge gaps may lie, and if these patterns have changed over time or vary across regions. Finally, we assessed what methodological approaches were leveraged in each paper to better understand any underlying epistemologies, problem framing, or approaches that may characterize a disciplinary approach. Importantly, our review focuses on research that explicitly frames its subject in terms of wildfire resilience, rather than the broader body of research addressing processes or outcomes that may contribute to resilience. Related concepts, including vulnerability, adaptation, preparedness, recovery, and mitigation, may address overlapping dimensions of wildfire resilience without using resilience terminology. Thus, our synthesis characterizes how resilience is explicitly conceptualized and operationalized in the literature, rather than providing a comprehensive review of all research relevant to wildfire resilience. We then use this information to assess if, where, and how disciplines are more comprehensive versus siloed in their evaluation of wildfire resilience to identify where isolation may limit actionable insights.

### Materials and methods

#### Identification of papers

We used Web of Science and PubMed to identify an initial corpus of papers using the intentionally general search term “wildfire resilience.” The Web of Science search was conducted on March 25, 2024 and updated on January 2, 2025. We augmented this dataset with an additional PubMed search performed on July 22, 2025. Collectively, these searches returned 2,510 results, of which 356 were duplicates between Web of Science and PubMed. We used the Preferred Reporting Items for Systematic Reviews and Meta-Analyses (PRISMA) approach,^29^ to identify the subset of studies included in the review (Figure 1). We first removed articles that (1) were not peer reviewed or (2) were duplicates, corrections, letters, or special issue forewords. We then retrieved all the remaining documents we could. Finally, we excluded papers that were (1) not about wildfire, (2) not about resilience, (3) published in 2025, or (4) not in English (to avoid misinterpretation or inaccuracy stemming from translating terminology and technical language) (*n* = 591). All papers were scored by a single individual. This resulted in a total of 1,563 papers.

**Figure 1:**
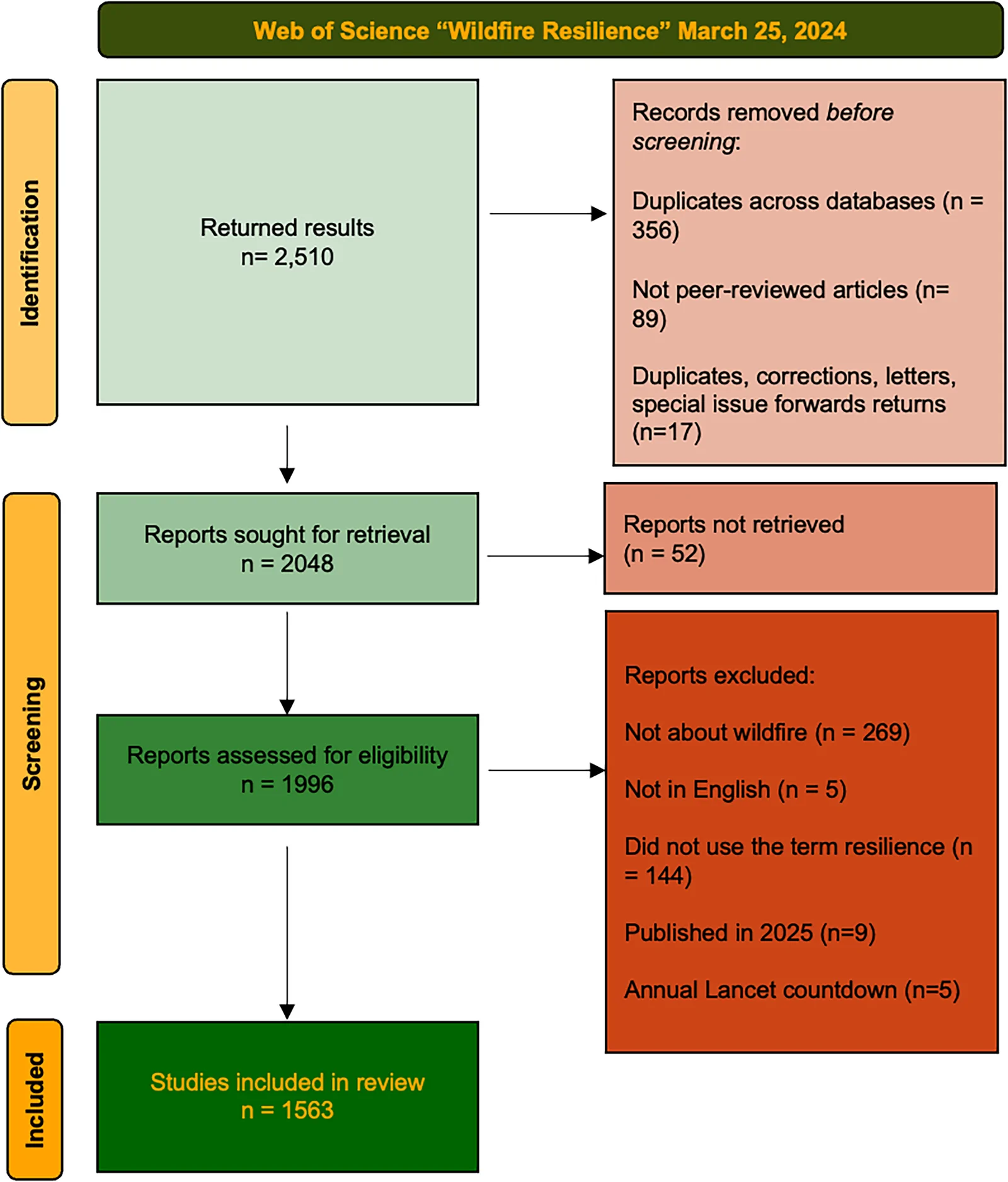
PRISMA workflow

#### Scoring

To evaluate patterns in the literature, we extracted information from each paper on country/ies of focus, social and/or ecological domain(s), methodology used to measure resilience, definition of resilience, and components of resilience that were tracked. Domain and methodological categories were defined prior to scoring and applied consistently across papers. Because wildfire resilience research often spans multiple disciplines and approaches, categories were not mutually exclusive; papers could be assigned to multiple domains and methodological categories when appropriate.

##### Country

For each paper, we listed the country or countries where the work was performed. For global studies, we did not list every country; these studies are therefore excluded from our assessment of geographic patterns of wildfire resilience, but are included in the remaining analyses.

##### Domain

Adapted from existing efforts^30^, we defined nine domains *a priori,* and then assessed which domain(s) each paper addressed. These domains were selected because they reflect dimensions commonly used to evaluate wildfire resilience in practice, including efforts such as the California Wildfire & Forest Resilience Task Force framework and the Wildfire Resilience Index (wildfireindex.org). Domains included: air, carbon, communities, habitats, infrastructure, livelihoods, sense of place, species, and water. Although not universally adopted, these domains capture most of what most people care about with respect to wildfire resilience. For example, these domains reflect existing efforts to quantify wildfire resilience in California.^31^ We do not necessarily expect these domains to be weighted equally, but nonetheless comparing relative amounts of effort is useful for exposing where particular emphases or biases may lie.

##### Methodology

To better understand the diverse methodological approaches used in wildfire resilience research, we scored techniques as: data science (e.g., analyses integrating large or heterogeneous datasets), experiment, field survey, modeling, remote sensing, survey (people), and/or synthesis/review. These categories were selected to capture the primary approaches used to generate, analyze, and synthesize information in wildfire resilience research. We intentionally did not classify studies into mutually exclusive methodological types because many studies combine approaches; instead, we evaluated the range of methods used within and across domains. Individual studies were therefore assigned to multiple methodological categories when appropriate.

##### Definitions

Resilience is a term used across multiple disciplines. To better understand how different studies define resilience, we recorded if a paper explicitly defined resilience, and if so, what reference was used to support the definition, if any.

##### Components

We evaluated which components of resilience, if any, each paper reported to measure, and categorized the study as addressing resistance, recovery, or both. We focused on resistance and recovery because these components are among the most consistently used dimensions of resilience. We recognize that resilience can also encompass additional dimensions, including adaptation, preparedness, redundancy, and resourcefulness; however, these concepts are variably defined across disciplines and were not consistently reported in the literature, making systematic classification challenging. When possible, we deferred to the classification provided by the author.

Because all papers were coded by a single reviewer, classification decisions may reflect individual judgment. However, predefined categories, consistent application of criteria across the dataset, and allowing multiple assignments for interdisciplinary studies helped minimize subjective classification.

## Results

Wildfire resilience research has grown rapidly in the last three decades, with the earliest publication in 1997 and over 240 papers published in 2024 (Figure 2A). Total papers published per year ranged from 1 to 248 (in 2023). Research on infrastructure resilience grew most rapidly (∼44% per year), while work on sense of place has not increased significantly over time; research on other domains grew 14%–22% per year. Research on wildfire resilience in habitats was the most common across the entire period (1997–2024), comprising more than half of all papers (Figures 2A and 3). The next most common domains were communities (16%) and species (13%). Sense of place and air were the least studied (less than 4% each) (Figure 3).

**Figure 2:**
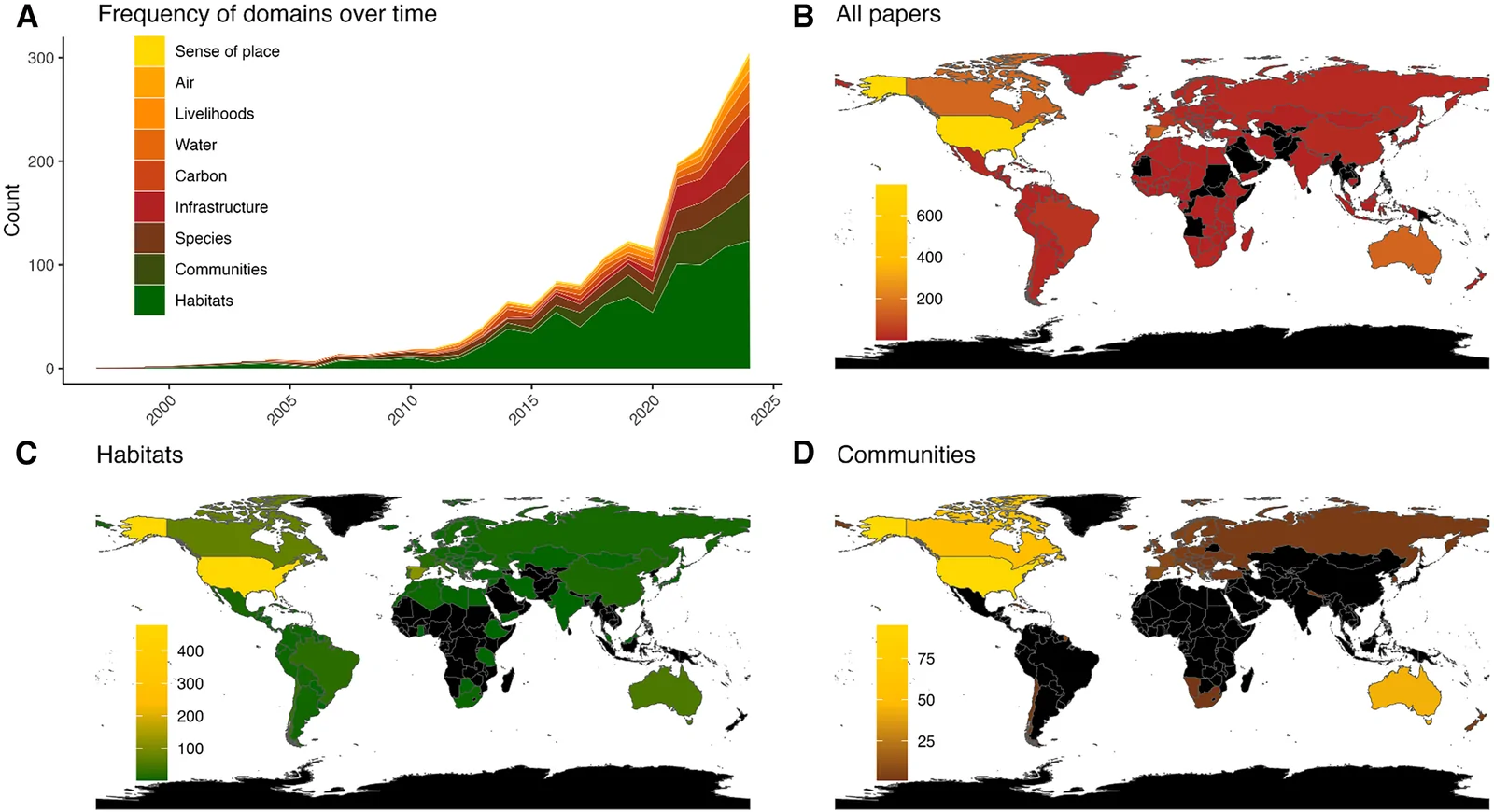
Number of publications has grown exponentially over time but unevenly across domains, with geographic concentrations (A) Number of papers addressing each of the nine domains (different colors). (B) Research covered 155 countries, with most studies in the United States. Spatial patterns were similar for the two most common domains, habitats (C) and communities (D). In each map, the scale bar indicates the number of studies, and black indicates an absence of papers. We did not include 14 global studies in this figure. Note difference in scale for each map.

**Figure 3:**
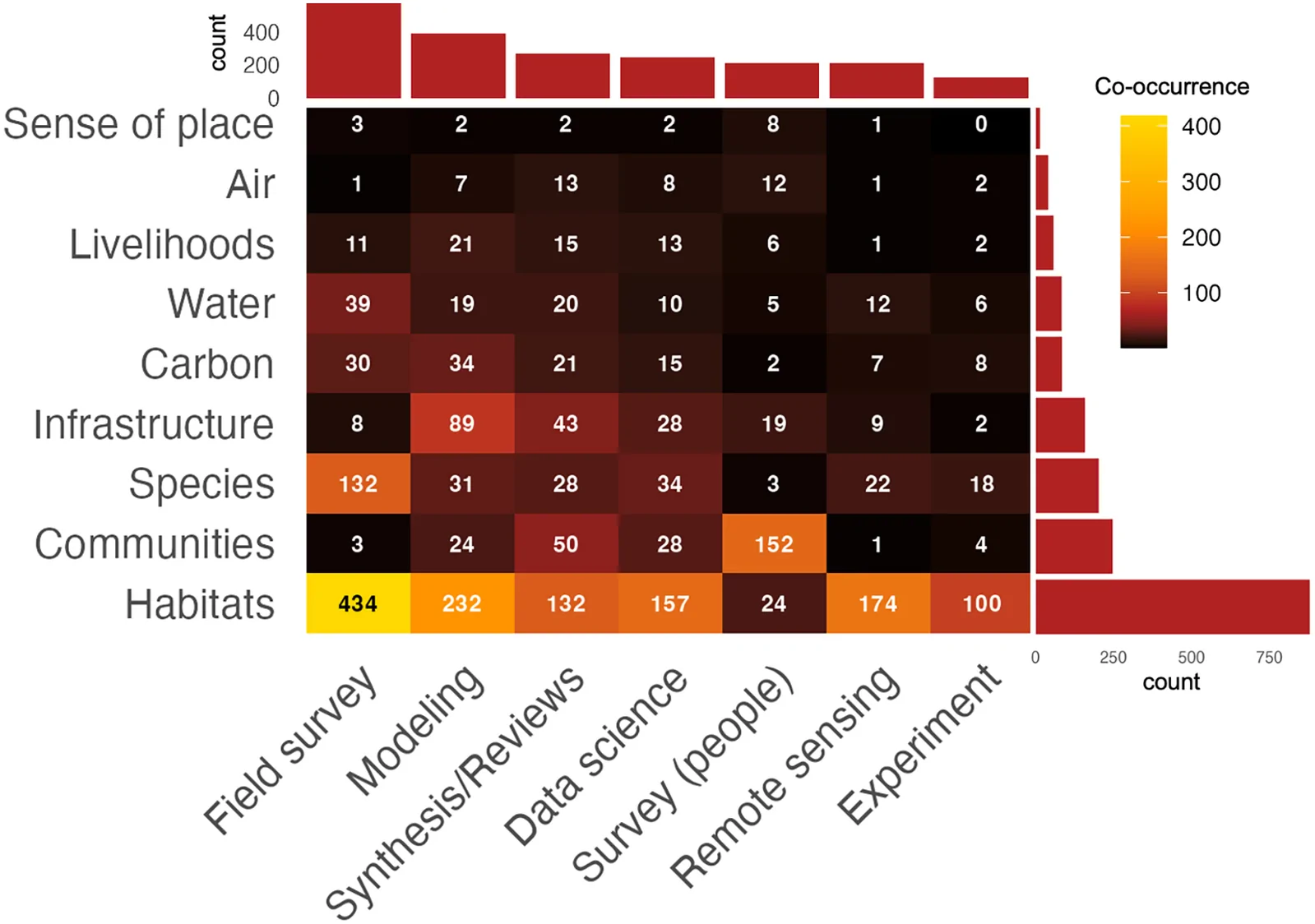
Bar charts and heatmap showing the frequency of methods (top) and domains (right) summed across all time periods and the co-occurrence of methods across domains (center grid) Brighter yellow colors show areas of concentration between methods and domains, while black regions indicate rarer combinations. Values in each grid cell indicate the number of studies for that combination; higher values indicate more frequent use of a methodological approach within a domain, whereas lower values indicate less common methodological-domain combinations.

We found strong publication bias toward western, well-developed, fire prone countries (Figure 2B). Most research has been conducted in the United States (*n* = 749), followed by Spain (*n* = 160), Canada (*n* = 150), Australia (*n* = 147), and Portugal (*n* = 56); all other countries had 50 or fewer papers. Some regions that have experienced substantial amounts of wildfire have had limited research on wildfire resilience (e.g., sub-Saharan Africa, Russia, and Amazonia). For papers that focused specifically on either habitats or communities (56% and 16% of total, respectively), we find a near absence of research in South America, Africa, and Asia (Figures 2C and 2D).

The variation in methods used to study different domains of wildfire resilience suggests potential biases and different ways of acquiring information (Figure 3). For example, surveys can help describe what is present, experiments can help explain why systems operate the way they do, and models can help predict or forecast what will happen under different management scenarios. We found that work on habitats, species, water, and communities most frequently used survey approaches, while research on infrastructure most commonly used modeling approaches. In contrast, several pairs of domains and methods rarely occurred, such as air, sense of place, or infrastructure with experiments, which may limit our understanding of a specific domain and highlight opportunities for alternate approaches to glean novel insights. For example, experiments in air quality research identified how to manufacture biochar while minimizing smoke creation,^32^ while an experiment in infrastructure evaluated structure design standards.^33^ Thus, the use of uncommon approaches within a domain has the potential to unlock unique and important information.

Most work focused on a single domain (79%), with no evidence of an increase in multi-domain work over time (Figure 4A), indicative of limited cross-cutting work. However, within the multi-domain papers, we found some more common and particularly uncommon pairings of domains (Figure 4B). For example, habitats were most frequently paired with species, a logical result given the tight dependencies of biodiversity on natural systems. Similarly, habitats were also strongly coupled to carbon, which is expected given the role of nature in the carbon cycle. In contrast, a less frequent combination was habitats and air, even though smoke originates from natural systems. Similarly, we find a low frequency of overlap between species and livelihoods, which contrasts recent efforts in other systems to financially evaluate biodiversity benefits.

**Figure 4:**
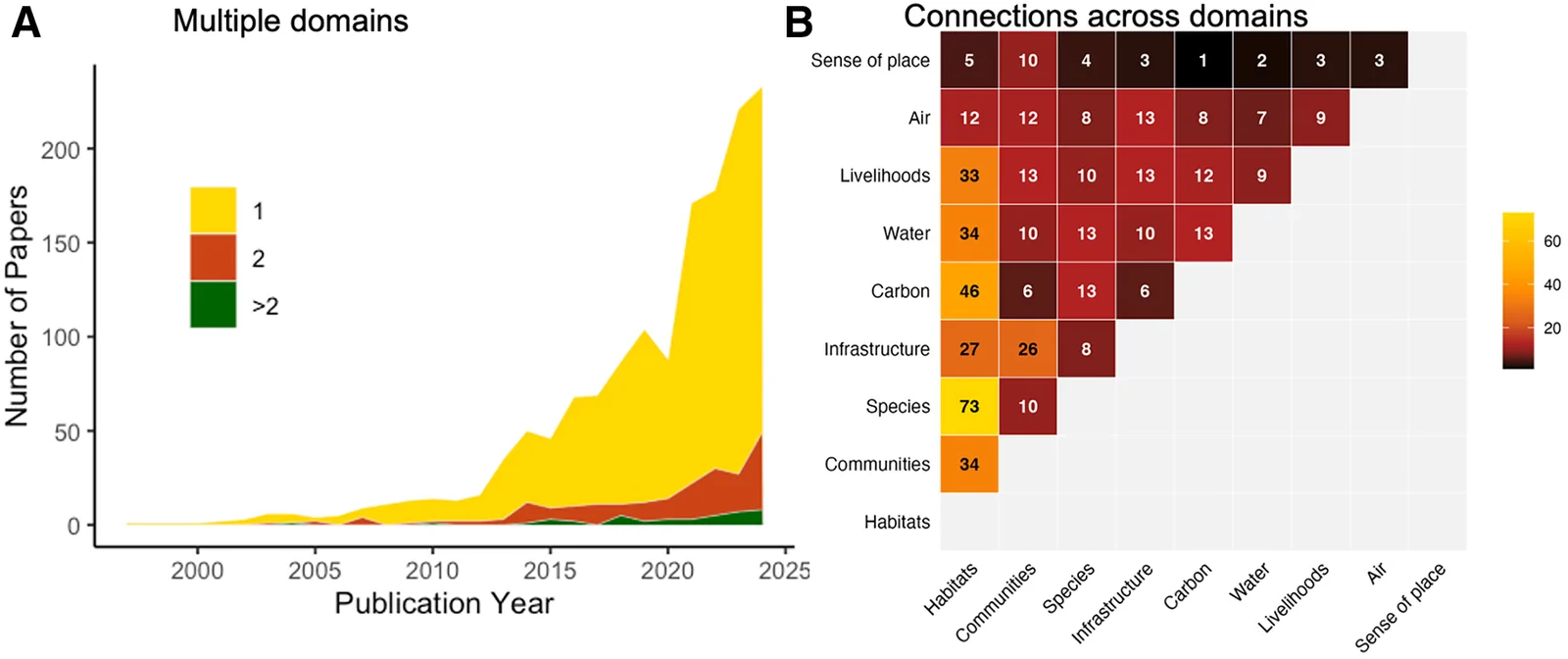
Frequency of multi-domain research (A) Number of papers addressing one, two, or more than two domains of the papers that included >1 domain (*n* = 225). (B) Heatmap showing the number of papers addressing pairs of domains. Although the presence of multiple domains within a study indicates potential interdisciplinary engagement, it does not capture the depth with which those domains are integrated.

While less than a quarter of papers actually defined resilience (*n* = 343, 21.9%), among those that did there was general consensus, with definitions broadly focused on resistance and recovery following Holling’s seminal 1973 paper and further refined by both Gunderson (2000) and Walker et al. (2004). Resistance describes a system’s ability to withstand or absorb a disturbance without significant change, while recovery describes the ability to return to the original state (or preferred state for adaptive or transformative resilience).^20^^,^^21^^,^^22^ Following these definitions, we categorized components of resilience as resistance and recovery for all papers. We found most papers (75%) addressed at least one component of resilience, typically addressing resistance or recovery; fewer papers (20%) addressed both (Figure 5). Among domains, different components of resilience were addressed. Specifically, for air, recovery was only evaluated in conjunction with resistance; otherwise, the rest of the research on air focused on resistance only. Similarly, resistance was the most frequently addressed resilience dimension for infrastructure. Finally, for both carbon and species, both components were most frequently considered together.

**Figure 5:**
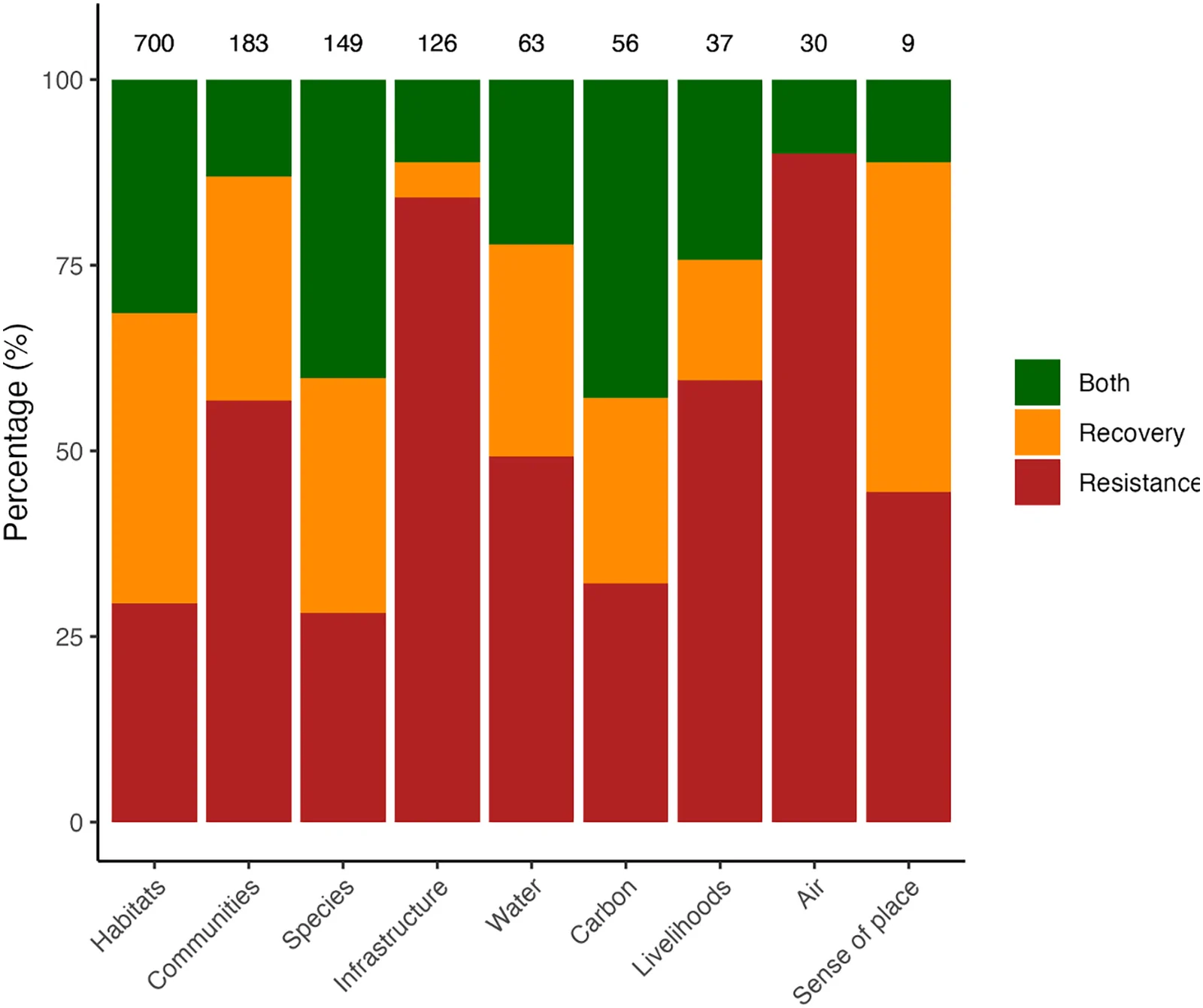
Patterns of evaluation of resilience components across the nine domains Total number of studies for the domain is shown at the top of each bar. Numbers cross-reference with Figure 1; 391 papers did not directly evaluate either component of resilience.

## Discussion

Resilience to wildfire is essential for living with it,^14^^,^^15^ yet key biases and gaps in research on wildfire resilience limit our ability to prepare and plan for wildfire across the diverse domains of public interest. The concentration of research in western, developed, fire-prone countries limits the transferability of results, likely more so for human- than nature-centric domains. Notably, these countries may have stronger institutions, more resources, and different governance structures, all of which may act as confounding factors that limit the transferability of findings to other contexts. For habitats, different regions will vary in species composition and community structure, yet resilience principles may be generalizable across pyromes—regions with shared fire frequency, intensity, size, area burned, and fire seasons—which are underpinned by biophysical settings and span continents.^1^^,^^2^^,^^3^ Thus, while the specific species may differ in North America and Africa, underlying ecological interactions and evolutionary processes shaping fire-adaptive communities might result in sufficient similarity to facilitate comparison, though this hypothesis warrants empirical testing. In contrast, for communities, results are likely less generalizable because human community structure, ties, and values are geographically variable.^34^ This bias is a particular concern given the paucity of research on wildfire resilience and communities in most countries.

Wildfire resilience research also remains siloed by discipline and practice, with no indication this pattern is changing. Calls to build cross-cutting wildfire resilience work are not new,^14^^,^^15^^,^^35^^,^^36^ yet most work remains confined to a single domain or single method, with each domain largely dominated by one or two methodological approaches. Thus, we may be missing out on key synergies and insights that could advance and transform our understanding of wildfire resilience. For instance, research integrating air (smoke exposure) and communities (mental health outcomes) highlights the significant negative mental health impacts of smoke exposure.^37^^,^^38^ Similarly, connecting wildfire resilience to carbon and livelihoods reveals co-benefits: selling low-value forest residues (e.g., woody fragments, chips, and lower quality logs) post-prescribed burn can incentivize fuel treatment while enhancing carbon sequestration,^39^ and converting mechanical thinning byproducts into biochar supports both economic activities and wildfire mitigation.^40^ Additionally, integrating infrastructure (prison design and structure) and communities (carceral social structure) research reveals the vulnerability of incarcerated populations to wildfires.^41^ These multi-domain efforts provide deeper insights into the cross-cutting nature and consequences of wildfire resilience.

Of all the domains, sense of place was the least well studied (*n* = 9), yet this domain captures the profound emotional and psychological bonds that people develop with specific landscapes. Wildfires can disrupt these bonds by altering or destroying familiar environments, leading to psychological impacts such as solastalgia or eco-anxiety.^42^^,^^43^^,^^44^ We argue that disruptions to sense of place can undermine resilience, particularly when individuals and communities are unable to adapt or reconnect. In such cases, the loss may represent a fundamental and potentially irreversible disconnection from the landscapes that shape identity, memory, and meaning. Given its importance and complexity, we identify sense of place as a critical research gap in the field of wildfire resilience, one that requires nuanced, interdisciplinary approaches to address effectively.

Air is the second most under-studied area of wildfire resilience, despite the significant negative health outcomes from and public attention to the issue. This paucity of research reflects the knowledge gaps on how communities and governments can build greater resilience to this substantial human health consequence of wildfires. Key gaps that need to be filled include reliable air quality data, causal linkages between smoke exposure and health outcomes, and research on the impact of repeated exposure. Recent estimates indicate wildfire smoke exposure results in >10,000 deaths in the United States annually,^45^ creating urgency to fill these gaps. In addition to strong evidence for negative effects on physical elements of health like cardiovascular and respiratory systems,^46^^,^^47^ emerging research indicates substantial negative mental health impacts from wildfire.^48^ Notably, research in this domain largely emphasizes the resistance component of resilience, likely reflecting a focus on minimizing or avoiding harmful health effects of smoke exposure. Such efforts to reduce harm are essential to living with fire as a natural and inevitable part of many landscapes. These negative outcomes may not be felt evenly across the general population; in the United States, some evidence suggests negative mental health outcomes may be disproportionately felt in rural areas, particularly among white men without a college education.^49^ The knowledge gaps in this domain meaningfully limit our ability to make informed policy decisions. For example, managers are often required to have smoke management plans for prescribed burns, a key mitigation strategy aimed at building wildfire resilience. Policy guidelines then rely on concentration thresholds to protect public health and safety, yet there is no truly “safe” level of smoke exposure. Smoke accompanies all wildfire, so addressing these gaps is essential for crafting comprehensive wildfire resilience strategies and reducing negative health outcomes.

We also found limited research on the resilience of infrastructure to wildfire, a surprising result given the emphasis on the built environment in wildfire resilience efforts (e.g., home hardening). Here, infrastructure resilience encompasses not only the structural resilience of buildings and other physical assets, but also the transportation, water-supply, firefighting, and other networked systems that support prevention, emergency response, evacuation, and recovery. One possible driver of this trend could be the focus on resistance rather than recovery strategies in the built environment, such that action is more focused on preventing loss, which may limit the use of the term “resilience” in the research. However, this pattern is beginning to change—infrastructure was the fastest-growing domain in the last few years. Indeed, there is substantial media, public, and policy attention around the wildfire crisis focused on the destruction of structures and loss of human life.^16^^,^^17^^,^^18^ As humans continue to develop, a better understanding of how to create structural resilience to wildfires is sorely needed, particularly elucidation of mechanistic linkages between particular actions and improved resilience.

Perhaps unsurprisingly, the majority of studies concentrated on habitats. Wildfires occur in natural landscapes, and most interventions to build resilience are focused on those landscapes. While wildfires have extensive impacts both within and outside of the fire perimeter—destroying structures, negatively impacting human health, and disrupting local economies-wildfires definitionally occur in natural habitats. Further, wildfires are a landscape process, and efforts to build resilience to wildfires typically occur at this scale, with common treatments including prescribed burns, mechanical clearing, and fuel breaks.^16^^,^^17^^,^^18^ Thus, research on the causes, consequences, and mitigation of wildfires logically occurs in the habitats domain.

The rarity of experimental studies on wildfire resilience limits our ability to uncover and explain mechanistic links. In the social and human health domains, this gap is understandable—human experiments are difficult, and the ones we identified were generally constrained to testing the efficacy of different interventions in assisting recovery.^50^ For natural systems, there may be ethical and logistical barriers hindering this type of work, for example, the difficulty of conducting controlled burns and the ethics of creating harmful smoke. However, there are interesting opportunities to be deliberate in how we study wildfire. Most notably, the recent growing interest in accelerating the pace and scale of prescribed burns offers an exciting opportunity to begin testing how to enhance resilience.^31^^,^^51^^,^^52^

As with all reviews, ours is necessarily constrained by scope, language, and methodology. We believe these limitations are important to recognize for proper interpretation; they do not, however, detract from the overall contribution of this work to understanding how wildfire resilience is conceptualized across the literature. First, we limited our analysis to English-language publications. As native English speakers, we determined that critically reviewing and accurately classifying technical literature in other languages would introduce a high risk of misinterpretation. Furthermore, most scientific literature is written in English. However, this decision biased our findings toward research conducted in English-speaking contexts and may have excluded valuable work published in other languages. This distinction is particularly important for regions such as Latin America, Africa, and parts of Asia, where relevant wildfire resilience research may exist in regional journals, institutional reports, or non-English literature not captured by our search strategy. Second, our search was restricted to the term “*wildfire resilience*.” While this is a relatively narrow search term, it was chosen to align with our research question, which focused on the conceptual use of wildfire resilience across disciplines. We recognize that related concepts, including wildfire adaptation, vulnerability, preparedness, recovery, and mitigation, may capture overlapping aspects of resilience and may not use the specific terminology of “wildfire resilience.” For example, research framed around wildfire risk has integrated natural and human systems using simulation modeling and remotely sensed data.^53^ This illustrates that relevant cross-domain research may exist outside the terminology captured by our search strategy.

Managing landscape-scale fire is a wicked problem: fire is both an inevitable natural process and a growing threat to human and ecological systems, especially as other anthropogenic pressures intensify across landscapes. Our management decisions over the last century have likely exacerbated this challenge, increasing wildfire debt and worsening outcomes from contemporary fires. At the same time, climate change is expected to further increase the frequency and severity of extreme wildfire events. Building resilience has therefore become a central strategy for reducing wildfire impacts on both people and ecosystems. However, as our review shows, the study and application of wildfire resilience remain fragmented and uneven across disciplines and geographies. Advancing this field will require more deliberate coordination, including shared conceptual frameworks, expanded methodological approaches, and interdisciplinary research that incorporates underrepresented domains and regions. Future research should increasingly examine how resilience across domains is interconnected, linking ecological, human, infrastructure, and other system responses to wildfire to better understand trade-offs, synergies, and pathways for resilience.

## Resource availability

### Lead contact

Requests for further information and resources should be directed to and will be fulfilled by the lead contact, Caitlin Fong (fong@nceas.ucsb.edu).

### Data and code availability

All data and all code required to perform the analyses and render the figures are available at github.com/WRI-Science/iScience_systematic_map.

## Acknowledgments

Thank you to the researchers, analysts, and staff at NCEAS for creating such an inspiring and collaborative place to work. We gratefully acknowledge funding and support from the 10.13039/100000936Gordon and Betty Moore Foundation (#11982) to B.S.H.

## Declaration of interests

The authors declare no competing interests.

## STAR★Methods

### Key resources table

**Table:** 

REAGENT or RESOURCE	SOURCE	IDENTIFIER
**Deposited data**		

Raw and analyzed data	This paper	Supplement

## Supplemental information

Data S1. Systematic literature review
